# Assessing the health benefits of development interventions

**DOI:** 10.1136/bmjgh-2021-005169

**Published:** 2021-02-16

**Authors:** Lucy S Tusting, Sandy Cairncross, Ramona Ludolph, Raman Velayudhan, Anne L Wilson, Steven W Lindsay

**Affiliations:** 1 Department of Disease Control, London School of Hygiene & Tropical Medicine, London, UK; 2 Centre on Climate Change and Planetary Health, London School of Hygiene & Tropical Medicine, London, UK; 3 Department of Environment, Climate Change and Health, World Health Organization, Geneva, Switzerland; 4 Veterinary Public Health, Environment and Vector Control, Neglected Tropical Diseases Headquarters, World Health Organization, Geneva, Switzerland; 5 Vector Biology Department, Liverpool School of Tropical Medicine, Liverpool, UK; 6 Department of Biosciences, Durham University, Durham, Durham, UK

**Keywords:** child health, environmental health, epidemiology, health policy

**Summary box**

- To achieve Sustainable Development Goal (SDG) 3—healthy lives and well-being for all people—development interventions (such as improved housing, water and sanitation) are critically needed in addition to biomedical interventions (such as drugs, vaccines and insecticides).
- However, many development interventions, such as house screening for vector-borne disease control, remain neglected in global health policy today.
- A major reason for this neglect is a requirement for evidence from rigorous systematic reviews and randomised controlled trials, which were designed for biomedical interventions and are poorly-suited to most development interventions.
- A new framework for assessing the health benefits of development interventions is urgently needed for the health sector to support and fully leverage the potential of the SDGs.

## Introduction

Biomedical interventions, such as therapeutics, vaccines and insecticides, are alone insufficient to achieve Sustainable Development Goal (SDG) 3—healthy lives and well-being for all ages. We also need development interventions to tackle the underlying determinants of ill-health by reducing deprivation and improving living conditions and the environment. This recognition formed the bedrock of early public health, from housing improvements and clean water provision in 19th century Europe and North America,^1^ to house screening for malaria elimination in the USA and water management for historical vector control in Italy, Sri Lanka, Panama and Zambia.^2^ Today, development interventions are a basic human right and ever more critical in response to rapid population growth, urbanisation and climate change.

Despite their importance, many development interventions remain neglected in global health policy. For example, the World Health Organization (WHO) recognises the need for multisectoral approaches to malaria,^3^ but based on current evidence is unlikely to be able to make a strong recommendation for strategies such as house screening and the removal of standing water to prevent mosquitoes in the environment. At the root of this issue lies a one-size-fits-all guideline development process designed for biomedical interventions, in which most development interventions fall short. Through rigorous systematic reviews and the GRADE process^4^ (often conducted by the Cochrane Collaboration; https://www.cochrane.org/), WHO bases guidelines on ‘high-certainty’ evidence, largely derived from randomised control trials (RCTs) where there is confidence that the true effect of an intervention is similar to the estimated effect.

## Why does health policy overlook development interventions?

Unfortunately, biomedical evidence assessments are ill-suited for evaluating the health effects of development interventions for three reasons. First, prioritisation of evidence from RCTs is inappropriate. Unlike vaccines and therapeutics, with homogeneous characteristics, effect size and feasibility, development interventions (eg, drainage to reduce malaria) are context-specific, necessitating numerous iterations of RCTs to generate an equivalent evidence base. RCTs of development interventions are themselves more challenging and expensive. Development interventions are often implemented over large areas (eg, installation of piped water). Estimation of a statistically robust effect size necessitates randomisation of an unfeasibly large number of areas. The interventions themselves can be complex and multifaceted (eg, malaria control in Khartoum uses land levelling and filling, water pipe repair and intermittent irrigation), complicating estimation of effect sizes. Blinding is impossible. Unlike biomedical interventions, development interventions have few financially viable pathways to scale-up. For all of these reasons, few RCTs of development interventions are done. This precludes meta-analyses, limits certainty of evidence and leads to calls for more RCTs or ‘paralysis by analysis’.

**Figure 1:**
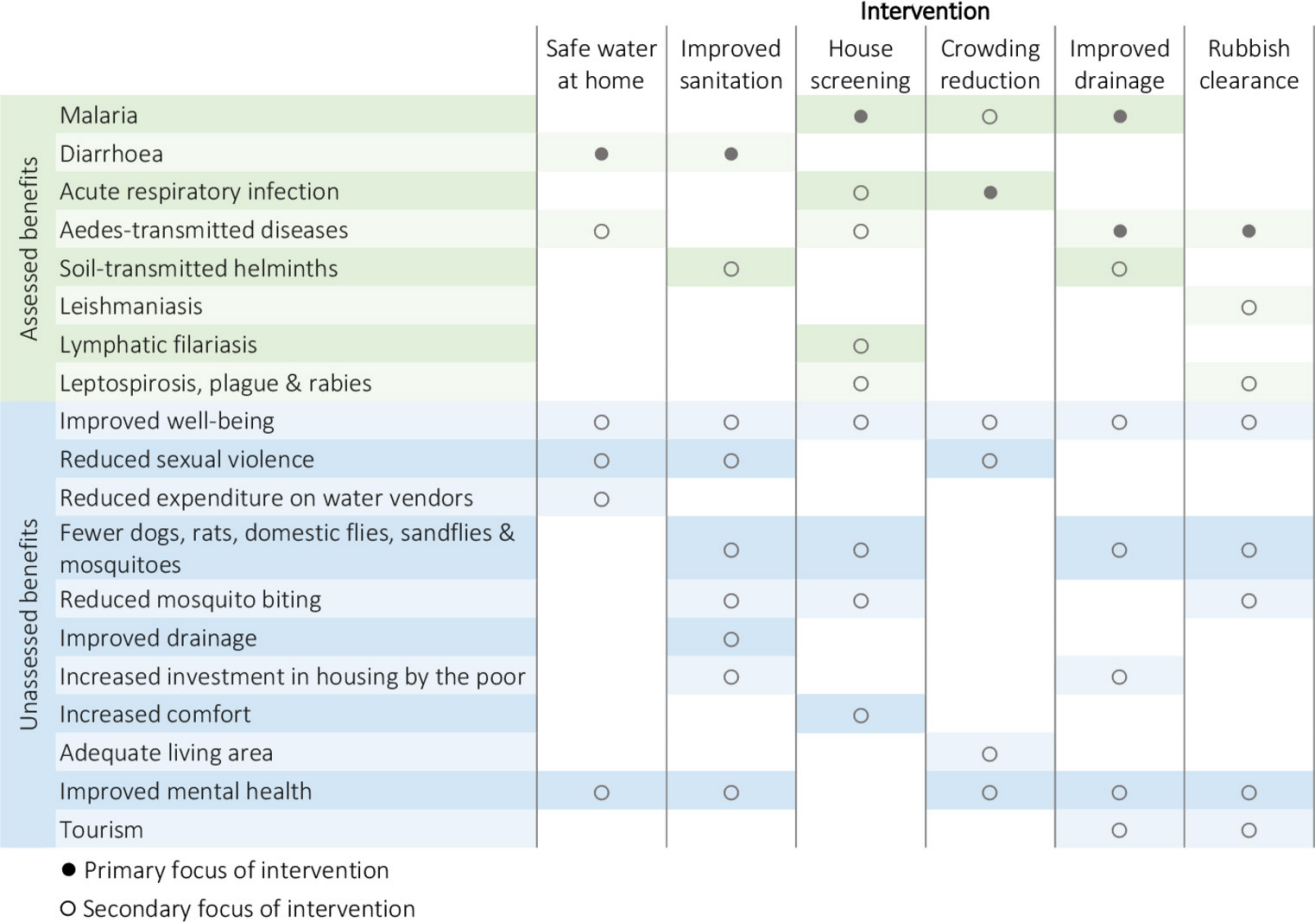
Co-benefits of development interventions.

Second, despite trends towards including other types of study, many systematic reviews still disregard historical, programmatic and observational evidence. The recent Cochrane review of housing improvements to prevent malaria, the oldest intervention against this disease, included only two studies of 4102 screened.^5^ Third, focusing systematic reviews on single health outcomes is logical for biomedical interventions, but misses the bigger picture for development interventions with wide co-benefits (figure 1).^6^ For example, a re-review of a Cochrane-standard systematic review of water, sanitation and hygiene interventions’ impact on child diarrhoea morbidity examined impacts within studies from a joint health and development perspective. Of 27 studies re-examined, 37% were judged to result in substantial additional impacts beyond reducing diarrhoea morbidity.^6^ Rather than narrow evaluations focused on single health outcomes, a better question is ‘How can we harness the health benefits of development interventions?’.

## Limiting health and development

Basing guideline development on biomedical evidence assessments alone is problematic for several reasons. First, taking a narrow view will impede global health goals by limiting our toolbox of interventions. Second, a system geared towards biomedical interventions will favour continued emphasis on vertical over horizontal health programmes among funders and decision-makers.^7^ Horizontal programmes, which are needed for most development interventions, take a holistic approach by strengthening health systems and community ownership over the long term,^7^ whereas vertical programmes, which are well-suited to delivering drugs, vaccines and insecticides, have specific, well-defined and usually quantitative objectives, centralised management and discrete resources.^8^ While national ministries of health are encouraged to adopt multisectoral and integrated approaches to disease control,^9^ vertical programmes are ultimately easier to fund, deliver and evaluate, leading to the promotion of targeted, technical solutions with clearly measurable outcomes.^10^ Expanding the guideline development process to be more inclusive of development interventions is a necessary step to ensuring a sustainable approach.

Third, a focus on biomedical evidence assessments is a lost opportunity for wider development goals. For example, clean water and sanitation are the core goals of SDG6. Both are also human rights since ‘everyone has the right to (an adequate) standard of living…’.^11^ Yet related Cochrane reviews found ‘insufficient evidence to know if source‐based improvements in water supplies… consistently reduce diarrhoea’^12^ and, for interventions to improve excreta disposal to prevent diarrhoea, ‘the quality of the evidence is generally poor and does not allow for quantification of any such effect’.^13^ Health guidelines reliant solely on such systematic reviews will constrain development for the poorest in society.^6^

## The way forward: a new approach to assess development interventions

We advocate the creation of a new approach to assess the health benefits of development interventions with three considerations. First, we must relax evidence criteria, accepting that RCTs may be logistically or ethically unfeasible. Non-randomised studies (including well-conducted before-and-after studies) must be considered, or systematic reviews will continue to find low-certainty evidence. Second, we must measure co-benefits of development interventions to avoid implementation delays from RCTs focused on single pathogens (figure). Third, we must consider the socioeconomic patterning of illness and opportunities to address these interlocking vulnerabilities. A step in the right direction is the new WHO INTEGRATE evidence-to-decision framework that, unlike GRADE, can evaluate complex interventions and assesses more criteria including human rights.^14^ But further refinement of this framework is needed alongside learning from other sectors. For example, systematic maps enable multiple forms of data to inform environmental policy and could supplement systematic reviews in health.^15^

## Conclusion

At this pivotal time, the health sector has a critical responsibility to leverage and support the potential of the SDGs, but cannot fulfil this within the current policy making framework. We urgently need a new framework for assessing the health benefits of development interventions to prevent further delays in their implementation and to maximise their impact.
